# Outcomes of Different Vital Pulp Therapy Techniques on Symptomatic Permanent Teeth: A Case Series 

**Published:** 2014-10-07

**Authors:** Saeed Asgary, Mahta Fazlyab, Sedigheh Sabbagh, Mohammad Jafar Eghbal

**Affiliations:** a *Iranian Center for Endodontic Research, Research Institute of Dental Sciences, Shahid Beheshti University of Medical Sciences, Tehran, Iran;*; b *Dental Research Center, Research Institute of Dental sciences, Shahid Beheshti University of Medical Sciences, Tehran, Iran *

**Keywords:** Calcium-Enriched Mixture, CEM Cement, Endodontic Treatment, Irreversible Pulpitis, Pulpotomy, Vital Pulp Therapy

## Abstract

In modern endodontics, vital pulp therapy (VPT) has been considered an ultra-conservative treatment modality. Based on the level of pulp preservation, VPT includes stepwise excavation, indirect pulp capping (IDPC), direct pulp capping (DPC), miniature pulpotomy (MP), partial/Cvek pulpotomy and coronal/complete pulpotomy (CP). The present article reviews the treatment outcomes of 94 permanent teeth with irreversible pulpitis treated with either IDPC (*n*=28), DPC (*n*=28), MP (*n*=29) or CP (*n*=9) using calcium-enriched mixture (CEM) cement. After a mean follow-up time of 12.3 months, 93 treated teeth were radiographic/clinically successful; only one radiographic failure was observed in the DPC group.

## Introduction

Although the value of a vital pulp in an immature permanent tooth is undeniable, its importance in a mature tooth cannot be overlooked [1]. Many authors have stated that the survival prognosis of endodontically treated teeth is not as good as teeth with vital pulps, which can be due to the loss of tooth structure as well as defensive mechanisms provided by the vital pulp such as tooth sensitivity and proprioception [2] as well as damping property [3]. 

There is no doubt that the biologic rationale for endodontic treatment is prevention or treatment of the only disease defined in this field, *i.e.* apical periodontitis (AP) [1, 4], which usually stems from a nonvital/infected pulp [1]. Therefore, it can be assumed that maintenance of the vital pulp ensures the prevention of AP and this is the paramount way of disease prevention [1, 4], keeping in mind that formation of AP around teeth with inflamed vital pulps is also possible [5].

There has been no universal agreement on the best treatment for cariously exposed vital pulps of permanent teeth [6]. While indication of mortal pulpectomy has several sensible reasons, vital pulpectomy gained general acceptance following several studies published before 1970s [4], with the rational being that the inflammation has probably reached a level where its elimination is not possible without removal of the entire pulp. The rationale for this treatment choice is first based on the unreliability of vital pulp therapy (VPT) on such teeth, which is challenged by recent high level of evidence (LoE) trials [7, 8, 9, 10] and second the high probability for success in cases of optimally-performed root canal therapy (RCT) on vital teeth [6, 11]. However, financial considerations [12] or low dental IQ [8], result in some patients refusing the optimal treatment. In other words, in many developing or even developed countries many patients cannot or do not want to afford such an extensive treatment on a tooth that shows clinical signs of irreversible pulpitis and ask for its extraction which undoubtedly is not the correct and ethical alternative treatment plan [7, 8, 12]. The most important issue is the impossibility of determining the reversible or irreversible nature of the pulpitis as a histological term [13], merely based on clinical sign/symptoms such as degree/characteristic of pain as they often do not reflect the pulp condition [13].

VPT of adults’ permanent dentition includes partial/miniature/coronal extirpation of the dental pulp (*aka.* pulpotomy) [14, 15] and covering the wound with biomaterials, or *in-situ* preservation of the whole pulp and its direct/indirect capping with the same biomaterials (*aka*. pulp capping) [16-18]. The ultimate goal of all these treatment modalities is preservation of the healthy portion of the pulp and therefore maintaining its healing ability [19, 20].

By keeping the infected/affected demineralized dentin, stepwise excavation/indirect pulp capping (IPC) decreases the risk of pulp exposure to caries and oral bacteria, and thus promotes a physiological reaction of the pulp-dentin complex [18]. The concept behind this treatment is sealing off the remnants of bacteria and inactivating them. The procedure provides an environment suitable for pulp healing process [21]. Direct pulp capping (DPC) involves treatment of the vital pulp exposure by sealing the pulpal wound by directly placing a biomaterial on the exposed pulp to facilitate formation of reparative dentin and maintain the vitality of the pulp [22]. DPC can be considered as one of the best treatments available for carious exposure of the vital pulps of permanent molars, when connection between the exposed pulp and oral cavity is sealed with appropriate biomaterials [18]. 

According to the guidelines of the American Association of Endodontists (AAE), partial/Cvek pulpotomy is the surgical removal of a small portion of the coronal pulp tissue to preserve the remaining coronal and radicular pulp. In other words the inflamed zone of the pulp is removed to the level of healthy noninflamed tissue [22]. Miniature pulpotomy (MP) was defined as the procedure with gentle/limited removal of the infected dentin chips/damaged pulp tissue specially the injured odontoblast cell layer after direct exposure of the pulp tissue that would not exceed ~1 mm; this treatment modality ensures a clean surgical wound and improved proximity/interaction of pulp covering agent to undifferentiated mesenchymal stem cells [23]. When it is assumed that the inflammation of the pulp tissue has extended to deeper levels of the coronal pulp, the entire pulp tissue is removed to the level of canal orifices (*i.e*. coronal pulpotomy-CP); afterwards hemostasis must be achieved and a biomaterial is placed over the remaining pulp tissue [22].

Experts are of the opinion that the success of VPT highly depends on *i*) the remaining pulp being either non-inflamed or capable of healing; *ii*) proper control of hemorrhage; *iii*) application of a biocompatible/regenerative capping material; and *iv)* presence of a bacterial-tight seal [6, 17, 18]. 

Various biomaterials, including mineral trioxide aggregate (MTA) [17, 24, 25] and calcium-enriched mixture (CEM) cement [7, 8, 26] have been proposed as capping agents for VPT. MTA appears to be particularly promising for placement in direct contact with pulp [24, 27, 28] as it induces dentin bridge formation while maintaining normal pulpal histology [27, 29]. Tooth discoloration, rather high cost and difficulty in handling are stated to be its main drawbacks [28]. 

CEM cement was introduced as a hydrophilic tooth-colored endodontic biomaterial with a composition different from MTA [30, 31]. CEM has favorable sealing ability and is biocompatible [32, 33], non-cytotoxic [27, 33, 34], and antibacterial [35, 36]. CEM is proved to be hard tissue inductive; dentinogenic [27, 34], cementogenic and osteogenic [32, 37-39]. All these properties make CEM a promising biomaterial for VPT cases.

Although VPT on traumatically exposed pulps proved to be very successful [1, 4, 16], some stated that VPT on the cariously exposed pulps may not be as predictable [16, 40]. There are a series of multi-centered randomized clinical trials that have assessed the one-, two- and five-year outcomes of VPT with CEM cement, on mature molars with signs/symptoms of irreversible pulpitis, that have shown radiographic and clinical success similar to one-visit RCT [7, 8, 10, 41, 42]. 

The present article focuses on the treatment outcomes of 94 permanent mature teeth from both genders treated with different modalities of VPT using CEM cement.

## Case Report

All treated teeth were diagnosed with irreversible pulpitis (prolonged lingering pain sensation upon stimulation with cold/hot stimuli). The selection of treatment was based on clinical judgment.

***1. Indirect pulp capping (IPC)***

Included in this report are 28 cases treated with IPC method. After local anesthesia with 2% lidocaine containing 1:80000 epinephrine, the teeth were properly isolated and caries were removed except for the affected unmineralized dentin covering the pulp surface, the removal of which would lead to pulp exposure. At this stage, the clean cavity walls were disinfected with a cotton pellet soaked in 5.25% NaOCl. CEM cement (BioniqueDent, Tehran, Iran) powder and liquid were mixed according to the manufacturer’s instructions. The creamy paste was placed over the pulpal wall(s) using a plastic instrument and packed with a dry cotton pellet. After 2-3 min, the cavities were permanently restored on the same session and the patients were put on a regular follow-up.

***2. Direct pulp capping (DPC)***

Similar to the previous group and under proper tooth isolation and local anesthesia, after pulp exposure of 28 teeth DPC with CEM was performed; all the caries in the cavity walls were removed and before pulp exposure the cavity was disinfected with a cotton pellet soaked in 5.25% NaOCl. The exposed pulp surface was not manipulated and bleeding (if any) was stopped with a sterile saline soaked cotton pellet. The rest of the procedure was conducted similar to the previous group.

***3. Miniature pulpotomy (MP)***

A total number of 29 cases were treated with this method. After local anesthesia and caries removal of the isolated teeth, cavity disinfection with 5.25% NaOCl and exposure of the pulp, the pulpal surface was gently shaved with brushing motion of a sterile #2 round diamond bur installed on a high speed handpiece accompanied with copious irrigation. After gaining hemostasis, the procedure of pulp covering with CEM cement and tooth restoration, followed the similar pattern.

**Table 1:** Treatment outcome in different treatment groups (IDPC: indirect pulp capping, DPC: direct pulp capping, MP: miniature pulpotomy, CP: coronal pulpotomy)

		**Treatment**				**Total**
		**IDPC**	**DPC**	**MP**	**CP**	
**Age**		31.0	32.6	32.8	27.8	31.7
**Gender**	**Female**	16 (57.1%)	17 (60.7%)	15 (51.7%)	5 (55.6%)	53 (56.4%)
	**Male**	12 (42.9%)	11 (39.3%)	14 (48.3%)	4 (44.4%)	41 (43.6%)
**Dental arch**	**Maxilla**	12 (42.9%)	17 (60.7%)	18 (62.1%)	6 (66.7%)	53 (56.4%)
	**Mandible**	16 (57.1%)	11 (39.3%)	11 (37.9%)	3 (33.3%)	41 (43.6%)
**Tooth type**	**Molar**	22 (78.6%)	23 (82.1%)	24 (82.8%)	7 (77.8%)	76 (80.8%)
	**Premolar**	6 (21.4%)	5 (17.9%)	4 (13.8%)	2 (22.2%)	17 (18.1%)
	**Incisor**	0 (0%)	0 (0%)	1 (3.4%)	0 (0%)	1 (1.1%)
**Filling material**	**Amalgam**	15 (53.6%)	12 (42.9%)	11 (37.9%)	6 (66.7%)	44 (46.8%)
	**Composite**	13 (46.4%)	15 (53.5%)	15 (51.8%)	3 (33.3%)	46 (48.9%)
	**Glass Ionomer**	0 (0%)	1 (3.6%)	3 (10.3%)	0 (0%)	4 (4.3%)
**Filling type**	**Cl I**	3 (10.7%)	2 (7.1%)	4 (13.8%)	1 (11.2%)	10 (10.6%)
	**Cl II**	18 (64.3%)	15 (53.6%)	14 (48.4%)	4 (44.4%)	51 (54.2%)
	**Cl IV**	0 (0%)	0 (0%)	1 (3.4%)	0 (0%)	1 (1.1%)
	**Cl V**	0 (0%)	0 (0%)	1 (3.4%)	0 (0%)	1 (1.1%)
	**Build-up**	7 (25.0%)	11 (39.3%)	9 (31.0%)	4 (44.4%)	31 (33.0%)
**Follow-up period**		11.4	13.4	11.9	12.6	12.3
**Success rate**		28 (100%)	27 (96.4%)	29 (100%)	9 (100%)	93 (98.9%)

***4. Coronal pulpotomy (CP)***

All the 9 cases treated with this method had similar treatment procedure; the caries was removed after local anesthesia and tooth isolation, the cavity was disinfected with 5.25% NaOCl and the pulp was excavated with a high speed round bur. Subsequently, the pulp surface was covered with CEM cement and the tooth was permanently restored. 

The Pearson chi-square test was used to assess the effect of patients’ gender, dental arch and tooth type on treatment success. Also for evaluating the relation of patients’ age and follow-up period with treatment success the one way ANOVA test was chosen. The level of significance was set at 0.05.

## Results

The details of the treated cases *i.e.* tooth type, restoration material and restoration type, are presented in Table 1. Different VPT treatments were done on incisors, premolars and molars. The mean follow-up duration for all treatment groups was 12.3 months and the average age of patients was 31.7 years old. The results revealed that treatment of only one case out of 94 was not successful while 93 other cases were vital and symptomless, showing a very high success rate of 98.9%. The failed case was in DPC group.

According to the results of the chi-square test, patients’ gender, dental arch (maxillary *vs.* mandibular) and tooth type did not have a significant effect on treatment success, with the *P*-values being 0.47, 3.06 and 0.58, respectively. Also the one way ANOVA test did not reveal any significant differences between the patients’ age and mean follow-up period with treatment success rate (*P*=0.67 and 0.49, respectively).

***Indirect pulp capping (IDPC); ***a total of 28 cases (16 female and 12 male patients) with mean age of 31 years were treated with this method. The mean follow-up period was 11.4 months and all cases had favorable outcomes (success rate=100%). 

***Direct pulp capping (DPC); ***DPC was performed for 28 patients with mean age of 32.6 years old (17 female and 11 male patients) and the cases were followed-up for an average of 13.4 months. The success rate for this treatment group was 96.4% with one case out of 28 requiring root canal therapy. 

***Miniature pulpotomy (MP); ***twenty-nine cases with mean age of 32.8 years old (15 female and 14 male patients) were treated with MP. After mean follow-up duration of 11.9 months, all cases (100%) had successful outcomes.

***Coronal pulpotomy (CP); ***all nine patients (5 female and 4 male patients) treated with this technique had successful outcomes (100% success rate). The mean follow-up time and mean age of patients were 12.6 months and 27.8 years, respectively. 

**Figure 1:**
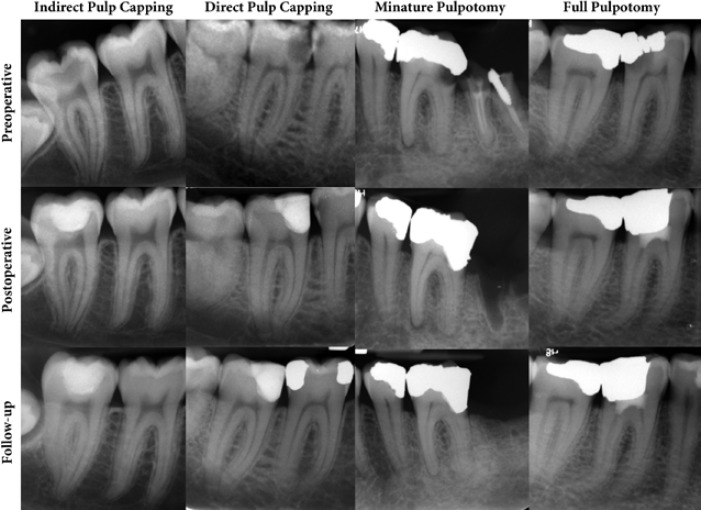
Preoperative, postoperative and follow-up radiographies of four mature molars treated with indirect pulp capping, direct pulp capping, miniature pulpotomy and coronal pulpotomy. In all cases, the widening of the periodontal ligament and periapical lucency is visible in the preoperative radiographs, which totally vanished in the follow-up clichés. Note that all cases were diagnosed with clinical signs of irreversible pulpitis

Figure 1 represents the preoperative, postoperative and follow-up radiographies of four cases treated with different VPT modalities.

## Discussion

This case series focused on treatment outcomes of different VPT modalities (*i.e.* IDPC, DPC, MP and CP) using CEM cement on 94 mature teeth, including mandibular/maxillary incisors, premolars and molars with irreversibly inflamed vital pulps. 

The dental pulp can be exposed by accidental trauma to a tooth, or by the dentist preparing a tooth for a restoration. The pulp typically is inflamed in either instance, because a mechanical exposure rarely occurs except during removal of a deep restoration or through overzealous excavation of deep caries [1, 17, 43]. However, VPT in mature permanent teeth has always been a matter of debate [6]. According to traditional school of thought, for a mature tooth with exposed vital pulp RCT is indicated [43], based on the unreliability of VPT on these teeth and the high probability for success if optimal RCT is performed [11]. While it is easy to write about optimal treatment, the fact remains that many patients do not want to or cannot afford such an expensive treatment (*i.e.* RCT) on a tooth that shows diagnostic or clinical signs of irreversible pulpitis. These patients may end up choosing tooth extraction [4, 6, 44]. If one puts all these factors alongside the undeniable unreliability of diagnosing irreversible pulpitis based on clinical criteria [13], prescribing RCT for all exposed pulps does not seem justified. Especially in modern endodontics where reproducing the vital pulp in necrotic teeth has become the top goal [45], extirpating the already existing vital pulp is not accepted anymore. 

Decision-making during management of clinical problems should be based on the best currently available evidence [6, 46]. Authors of the current study have been working on a randomized clinical trial to compare the treatment outcomes of permanent-teeth pulpotomy with sign/symptoms of irreversible pulpitis using CEM cement. The one-, two- and five-year results have been evaluated and reported [7, 8, 10]. Randomized clinical trials with long term follow-ups and large sample sizes are graded the highest rank in LoE pyramid [46]. The aforementioned trials revealed that not only VPT of mature teeth with CEM cement is not inferior to RCT, but also considering the high cost implemented on patients by failed RCTs (*i.e.* potential tooth extraction after probable procedural mishaps), VPT can be considered as a more valid treatment strategy [7, 8, 10]. Moreover the aforementioned trials were done by 23 calibrated dentists, whilst all cases in this study were treated by one endodontist. This fact can justify the higher success rates reported in this study. It is also worth mentioning that almost all high LoE studies in this regard have assessed coronal pulpotomy (CP) and have reported successful outcomes [6, 7]. However, this treatment is the most aggressive modality of VPTs and has two shortcomings: impossibility of further follow-up of the pulp status with the vitality tests and difficultly, if not impossibility, of conventional RCT in case of treatment failure since the canal orifices are obstructed with capping material [22]. A multi-center randomized controlled trial has compared the success of CP in mature permanent teeth with ProRoot MTA (*n*=208) and CEM cement (*n*=205). The clinical and radiographic success rates for MTA at 12-month follow-up were 98% and 95% while for CEM cement they were 97% and 92%, respectively. The difference between clinical and radiographic outcomes was not significant [7]. In addition, VPT with CEM cement can be considered as a suitable treatment option for patients suffering from symptoms of irreversible pulpitis because it reduces pain more effectively than RCT [41]. In this report, except for 9 cases treated with CP with success rate of 100%, IDPC (*n*=28), DPC (*n*=28) and MP (*n*=29) were also done with 100% success rate for all modalities except for DPC that was 96.4% successful. This can be a preliminary study for designing future randomized clinical trials with high LoE for defining non/less aggressive VPTs. 

In Iran, most of the RCTs are carried out by general dentists; therefore, iatrogenic errors may occur which reduce the longevity of treated teeth [44, 47]. As a more affordable treatment, VPT offers the advantage of requiring less specialist apparatus and materials, and therefore may have huge social and economical rationalization in both developing and developed nations. Additionally, in VPT radiography is not compulsory but advisable for patient’s follow-up. This means that the VPT has easier accessibility than RCT; however, the inability to retreat these teeth in cases of failure has not yet been reported meaning that in case of VPT failure the RCT is still an available option [10].

Considering the results of the current study, patients’ age, tooth type/position did not implement any effect on treatment success. This is not the mentioned issue in many previous studies, especially for age [1, 4, 19, 20, 22]. Moreover, according to a recent systematic review, VPT should be considered as an alternative treatment to pulpectomy in vital permanent teeth with carious pulp exposure, and partial/full pulpotomy is more predictable than DPC [6]. 

The last but not least is the undeniable role of tooth restoration and sealing which can imply support for the ongoing healing in the pulp by providing a bacteria-free environment [48]. Apart from the VPT *per se*, tooth restoration and its quality guarantees the successful outcomes of the treatment and the results of the reported cases confirm this fact.

## Conclusion

The various forms of vital pulp therapy can be considered very successful in meticulously chosen cases, provided that criteria such as perfect isolation, atraumatic procedure for the pulp, pulp covering with a perfect biomaterial, high quality restoration and patient management, are met.
